# ERK2 mediates inner hair cell survival and decreases susceptibility to noise-induced hearing loss

**DOI:** 10.1038/srep16839

**Published:** 2015-11-18

**Authors:** Takaomi Kurioka, Takeshi Matsunobu, Yasushi Satoh, Katsuki Niwa, Shogo Endo, Masato Fujioka, Akihiro Shiotani

**Affiliations:** 1 Department of Otolaryngology, National Defense Medical College, Saitama, Japan; 2 Department of Anesthesiology, National Defense Medical College, Saitama, Japan; 3 Aging Neuroscience Research Team, Tokyo Metropolitan Geriatric Hospital and Institute of Gerontology, Tokyo, Japan; 4 Department of Otolaryngology, Keio University School of Medicine, Tokyo, Japan

## Abstract

Extracellular signal-regulated kinase (ERK) is a member of the family of mitogen-activated protein kinases (MAPKs) and coordinately regulates a multitude of cellular processes. In response to a variety of extracellular stimuli, phosphorylation of both threonine and tyrosine residues activates ERK. Recent evidence indicates that ERK is activated in response to cellular stress such as acoustic trauma. However, the specific role of ERK isoforms in auditory function is not fully understood. Here, we show that the isoform ERK2 plays an important role in regulating hair cell (HC) survival and noise-induced hearing loss (NIHL) in mice (C57BL/6J). We found that conditional knockout mice deficient for *Erk2* in the inner ear HCs had hearing comparable to control mice and exhibited no HC loss under normal conditions. However, we found that these knockout mice were more vulnerable to noise and had blunted recovery from NIHL compared to control mice. Furthermore, we observed a significantly lower survival rate of inner hair cells in these mice compared to control mice. Our results indicate that ERK2 plays important roles in the survival of HC in NIHL.

Noise-induced hearing loss (NIHL), a major form of sensorineural hearing loss (SNHL), is thought to result primarily from damage to cells of the neurosensory epithelium (NSE), such as inner and outer hair cells (IHCs and OHCs), as well as to supporting cells (SCs). Since the mammalian NSE is unable to replace lost sensory cells, sensorineural deafness due to loss of these cells is irreversible^1^,^2^,^3^,^4^,^5^. However, the underlying mechanism of acoustic trauma is not precisely understood.

Extracellular signal-regulated kinases (ERKs) are members of the mitogen-activated protein kinase (MAPK) family. ERKs are activated by phosphorylation in response to a variety of extracellular stimuli and mediate diverse physiological processes such as cell survival, protein synthesis, cell proliferation, growth, migration, and apoptosis^6^. Accumulating evidence indicates that in response to acoustic trauma, ERK1/2 is phosphorylated in the hair cell (HC)^7^,^8^. However, the function of ERK activation in the HC in response to acoustic trauma is controversial: it is largely unknown whether activated ERKs promote or protect from noise-induced HC death. It is well known that ERKs are involved in cell death^9^,^10^. For example, in the NSE it has been reported that MAPK/ERK kinase1/2 (MEK1/2) inhibition significantly reduces IHC death caused by 8 h of neomycin treatment, suggesting that activation of ERK in SCs might promote HC death^11^. On the contrary, some studies reported that active forms of ERK act as prosurvival factors^12^,^13^,^14^. For instance, long-term blockade (~48 h) of MEK1/2 causes HC death, suggesting that basal levels of ERK activation in HCs is important for their survival^13^.

Recently, some reports have suggested that isoforms of ERK, ERK1, and ERK2 may play distinct roles in some conditions, although these isoforms share 84% of amino acid identity and have a very similar substrate profile^15^. Nonetheless, the relative roles of ERK1 and ERK2 in the HC in response to acoustic trauma remain largely unknown. To analyze ERK signaling, researchers frequently use an inhibitor of the upstream kinase MEK. Nevertheless, since ERK1 and ERK2 are both activated by MEK, inhibiting MEK will inhibit both ERKs, rendering it difficult to ascribe each isoform a unique physiological function. To circumvent this issue, we examined the role of ERK2 in the cochlea using HC-specific *Erk2* conditional knockout mice (hereafter referred to as HC-E2CKO). We found that auditory thresholds of HC-E2CKO mice were significantly increased compared to control mice after noise exposure, suggesting that ERK2 plays an important role in protecting the inner ear from noise exposure. To investigate the mechanism underlying the increased auditory thresholds of HC-E2CKO mice after noise exposure, we investigated the death of HCs. We also investigated the changes of synaptic ribbons of IHCs since hearing relies on synaptic transmission at these synapses, although the function of synaptic ribbons is still largely unknown^16^,^17^,^18^. Our results indicated that, in response to acoustic trauma, ERK2 plays an important role in mediating the survival of IHCs and synaptic ribbons.

## Results

### Acoustic trauma increases phosphorylation of ERK1/2 in the cochlea

**Figure 1:**
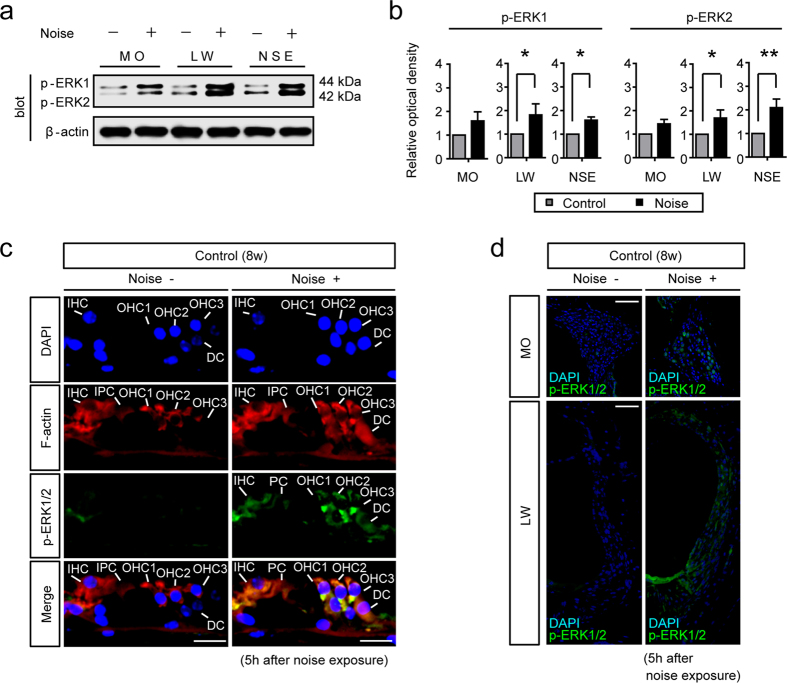
The phosphorylation levels of ERK1 and ERK2 are increased after noise exposure. (**a**) Representative western blots for the phosphorylation levels of ERK1 and ERK2 in the modiolus (MO), lateral wall (LW), and neurosensory epithelium (NSE) 5 h after noise exposure. (**b**) The phosphorylation levels of ERK1 and ERK2 (**b**) in the cochlea were upregulated 5 h after noise exposure. Specifically, phosphorylation levels of ERK1 and ERK2 in the LW and NSE were significantly upregulated 5 h after noise exposure (n = 4 for each group). β-actin served as the control for protein loading. Error bars show SE. (Mann-Whitney U-test, *p < 0.05, **p < 0.01). (**c**) In naive mice, p-ERK1/2 was not observed in the organ of Corti. By contrast, at 5 h after noise exposure, p-ERK1/2 was detected in IHCs, OHCs, and SCs, including IPCs and DCs. IHC,; inner hair cell; OHC, outer hair cell; SC, supporting cell; IPC, inner pillar cell; DC, Deiter’s cell. Scale bar, 20 μm. (**d**) In naive mice, p-ERK1/2 was not observed in the MO and LW. By contrast, p-ERK1/2 was detected in spiral ganglion neurons (SGNs) and spiral ligaments of the LW. Scale bar, 50 μm.

Phospho-ERK1/2 (p-ERK1/2), an active form of ERK1/2, has reportedly been detected in the spiral ligament and the NSE of the cochlea with peaks at 3–6 h after noise exposure^8^. Similar to the above study, we observed that 5 h after noise exposure, phosphorylation levels of ERK1 and ERK2 were increased in the modiolus (MO), lateral wall tissue (LW), and NSE (Fig. 1a,b) (n = 4 for each group). The quantification indicated that phosphorylation levels of ERK1 and ERK2 at the end of the 5 h period of noise exposure were significantly increased from those before noise exposure in the LW (Mann-Whitney U-test, ERK1, p = 0.018; ERK2, p = 0.036) and the NSE (Mann-Whitney U-test, ERK1, p = 0.018; ERK2, p = 0.004). Additionally, in order to detect p-ERK1/2 localization in the NSE, MO, and LW, we performed immunohistochemical analysis for p-ERK1/2 in the cochlea from naive and noise-exposed mice (Fig. 1c,d). In naive mice, p-ERK1/2 was hardly observed in the NSE, MO, and LW. On the other hand, in noise-exposed mice, p-ERK1/2 was detected in the NSE, such as in IHCs, OHCs, inner pillar cells (IPCs), and Deiter’s cells (DCs). We also detected p-ERK1/2 in the spiral ganglion neurons (SGNs) and spiral ligaments of noise-exposed mice (Fig. 1d).

### Cochlear morphology and expression levels of ERK1 are normal in HC-E2CKO mice

In this study, we used a *pou4f3* promoter-driven Cre transgenic mouse line, in which Cre activity is confined to the inner ear HC^19^,^20^, bred with *Erk2*^flox/flox^ mice. Transcripts for *pou4f3* are abundant in the developing and mature HC^21^. The resulting HC-E2CKO (*pou4f3-cre*+: *Erk2*^flox/flox^) mice were viable and fertile with normal appearance. The controls used for this study had one of the following genotypes; *pou4f3-cre*−*; Erk2*^+/+^, *pou4f3-cre*−*; Erk2*^flox/flox^, *pou4f3-cre*+*; Erk2*^+/+^ (hereafter termed control mice). We did not detect any differences among the different genotypes of the control group and we assumed that any phenotypic differences between them would be minimal.

**Figure 2:**
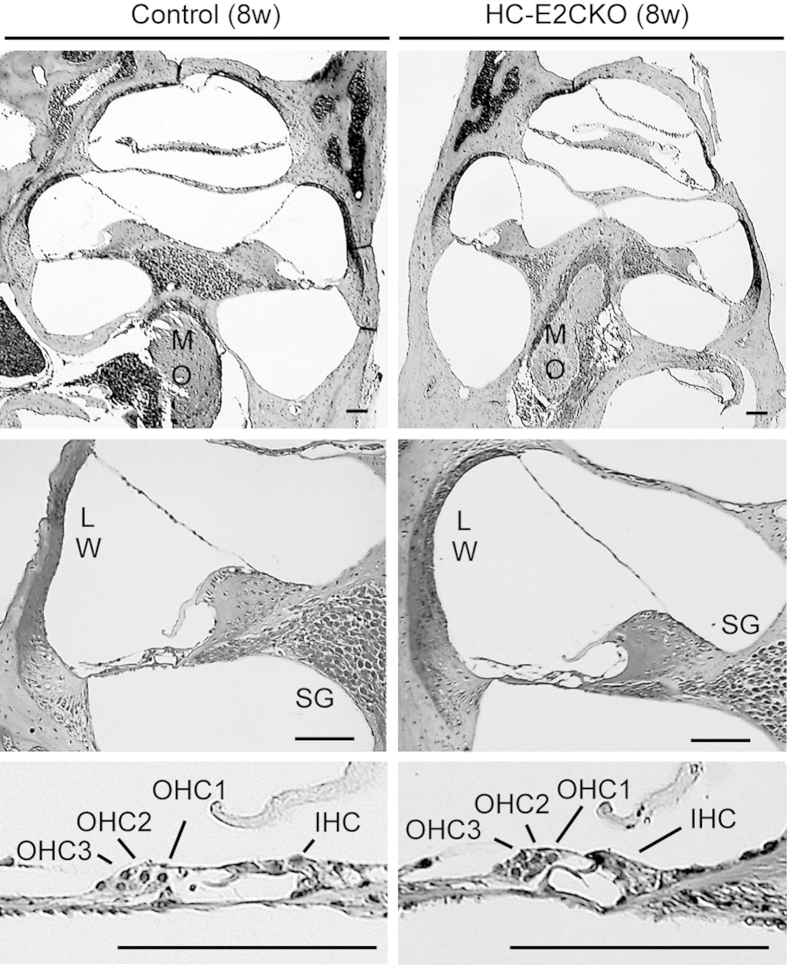
Cochlear morphology was normal in HC-E2CKO mice. Representative photomicrographs of cochleae stained with hematoxylin and eosin from 8-week-old control and HC-E2CKO mice. No significant difference was detected in the cochleae, including the lateral wall (LW), spiral ganglion (SG) in modiolus (MO), and inner and outer hair cells (IHCs, OHCs). Scale bar, 50 μm.

**Figure 3:**
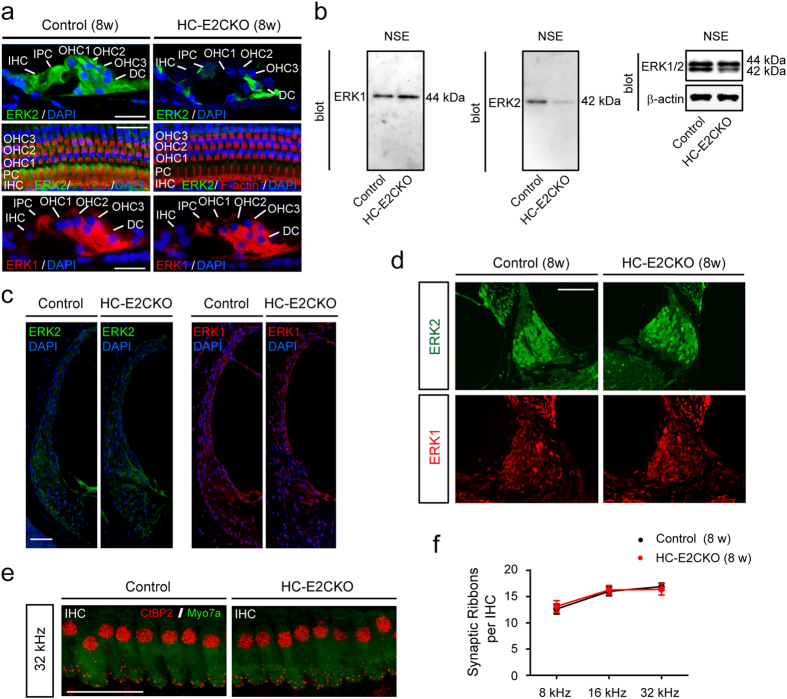
ERK2 expression was abrogated in the HC of HC-E2CKO mice. (**a**) Whole mount preparations and cross sections of cochleae from HC-E2CKO and control mice. IHCs, OHCs, IPCs, and DCs showed ERK2 immunoreactivity in control cochleae while HC-E2CKO cochleae exhibited no staining in HCs and weak staining in SCs. ERK1 expression was observed in OHCs and SCs and was unchanged in both HC-E2CKO and control mice. Scale bar, 20 μm. (**b**) Representative western blots of the NSE from control and HC-E2CKO mice. The 44-kDa (Fig. 3b, left) and 42-kDa (Fig. 3b, middle) bands were detected using anti-ERK1 and anti-ERK2 antibodies, respectively. The 42-kDa band from HC-E2CKO mice was reduced compared to that of controls (Fig. 3b, right). (**c**,**d**) No prominent difference in ERK1 and ERK2 expression was observed in the lateral wall (**c**) and spiral ganglion (**d**) between control and HC-E2CKO mice. Scale bar, 50 μm. (**e**,**f**) Whole mount preparations were stained with myosin7a (green) and CtBP2 (red) (e). Scale bar, 20 μm. No prominent difference in the number of synaptic ribbons was observed at any frequency (n = 4 for each group) (**f**). IHC, inner hair cell; OHC, outer hair cell; IPC, inner pillar cell; DC, Deiter’s cell, SC, supporting cell; Myo7a, myosin7a. Data represent mean values; error bars show SE.

To investigate the cochlear morphology of HC-E2CKO mice (8 weeks of age), we used hematoxylin-eosin staining to perform a histopathological study (Fig. 2). The cochleae of HC-E2CKO mice at 8 weeks of age appeared normal and there was no sign of HC and/or SGN loss. Staining for ERK2 in cross sections and whole mount preparations of the cochleae confirmed the abrogation of ERK2 in the HC of HC-E2CKO mice. In cochleae from control mice, ERK2 was prominent in IHCs, OHCs, and SCs, including IPCs and DCs (Fig. 3a). On the other hand, ERK2 expression was below detectable levels in all HCs in cochleae from HC-E2CKO mice (Fig. 3a). Moreover, HC-E2CKO cochleae exhibited reduced ERK2 expression in SCs, including DCs. However, the numbers and appearances of OHCs and IHCs in HC-E2CKO mice were comparable to control mice. Moreover, the expression of ERK1 in OHCs was unchanged in HC-E2CKO mice compared with control mice (Fig. 3a), indicating that conditional knockout of *Erk2* led to no compensatory changes in ERK1 expression. In contrast, ERK1 expression was below detectable levels in the IHCs of HC-E2CKO and control mice. We confirmed the specificity of ERK1 and ERK2 antibodies (Fig. 3b). Moreover, we investigated ERK1/2 expression in the LW and SGNs from control and HC-E2CKO mice (Fig. 3c,d). We observed no significant differences in the expression of ERK2 in the LW and SGNs between HC-E2CKO and control mice. Similarly, ERK1 expression was not changed in the LW and SGNs of HC-E2CKO and control mice.

We next investigated whether the number of synaptic ribbons was changed in HC-E2CKO mice (8 weeks of age) compared with control mice (n = 4 for each group). Cochlear IHCs transmit acoustic information to spiral ganglion neurons through synaptic ribbons, organelles found at presynaptic active zones of bipolar neurons that generate sustained graded electrical signals in response to stimuli^22^. Immunostaining for the transcriptional cofactor c-terminal-binding protein-2 (CtBP2), a synaptic ribbon structural protein, indicated that the number of synaptic ribbons per IHC was similar between HC-E2CKO and control mice before noise exposure (Fig. 3e,f).

**Figure 4:**
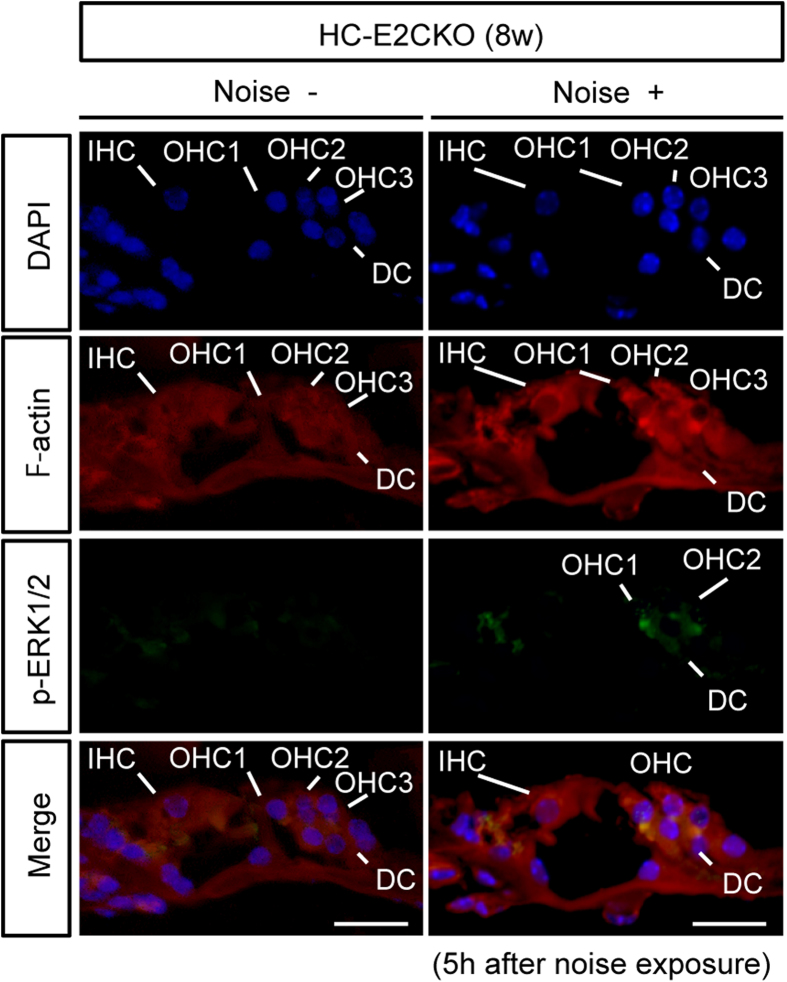
p-ERK1/2 was detected in OHCs and SCs but not in IHCs after noise exposure in HC-E2CKO mice. In naive HC-E2CKO mice, p-ERK1/2 was not detected in the organ of Corti. By contrast, at 5 h after noise exposure in HC-E2CKO mice, the immunoreactive signal of p-ERK1/2 was detected in OHCs and DCs, but not in IHCs. IHC; inner hair cell. OHC, outer hair cell; DC, Deiter’s cell. Scale bar, 20 μm.

To investigate the localization of p-ERK1 and p-ERK2, we performed an additional immunohistochemical study using an antibody against both pERK1 and p-ERK2 in HC-E2CKO mice after noise exposure (Fig. 4). We found that the immunoreactive signal of p-ERK1/2 increased in OHCs and DCs, but not in the IHCs, of HC-E2CKO mice after noise exposure. Because ERK2 was deleted in the OHCs as well as IHCs of HC-E2CKO mice, the increase in p-ERK signal of OHCs in HC-E2CKO mice after noise exposure might indicate the presence of p-ERK1.

### ERK2 contributes to the recovery of hearing following noise exposure

**Figure 5:**
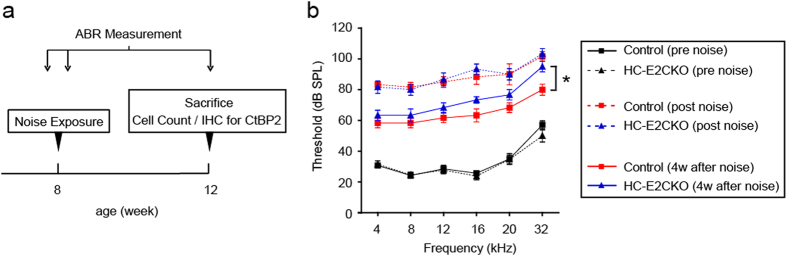
HC-E2CKO mice exhibited impaired recovery of ABR thresholds after noise exposure. (**a**) Experimental procedure. (**b**) Time course of changes in the ABR threshold before and immediately after noise exposure (pre and post), and then at 4 weeks after noise exposure are shown. Differences in the threshold 4 weeks after noise exposure between control and HC-E2CKO mice at 32 kHz (asterisk) were significant (n = 6 in each group). Data represent mean values; error bars show SE (Mann-Whitney U-test, *p < 0.05).

To examine the effect of ERK2 abrogation on NIHL susceptibility, we examined the response of HC-E2CKO mice to acoustic trauma (8 weeks of age) (Fig. 5a; n = 6 for each group). To induce severe damage to the HCs, animals were exposed to two octave-bands of noise (2–8 kHz) at a sound pressure level (SPL) of 121 dB for 5 h. The ABR thresholds before noise exposure were essentially equivalent in HC-E2CKO and control mice (8 weeks of age) at any frequency. The time course of the change in ABR threshold after noise exposure is shown in Fig. 5b. At 32 kHz, ABR thresholds of HC-E2CKO mice were significantly increased compared with the control group at 4 weeks after noise exposure (Mann-Whitney U-test, p = 0.025), suggesting that HC-E2CKO mice are more vulnerable to noise and may have less capacity to recover from NIHL than control mice.

### IHC loss was significantly increased in HC-E2CKO mice after noise exposure

**Figure 6:**
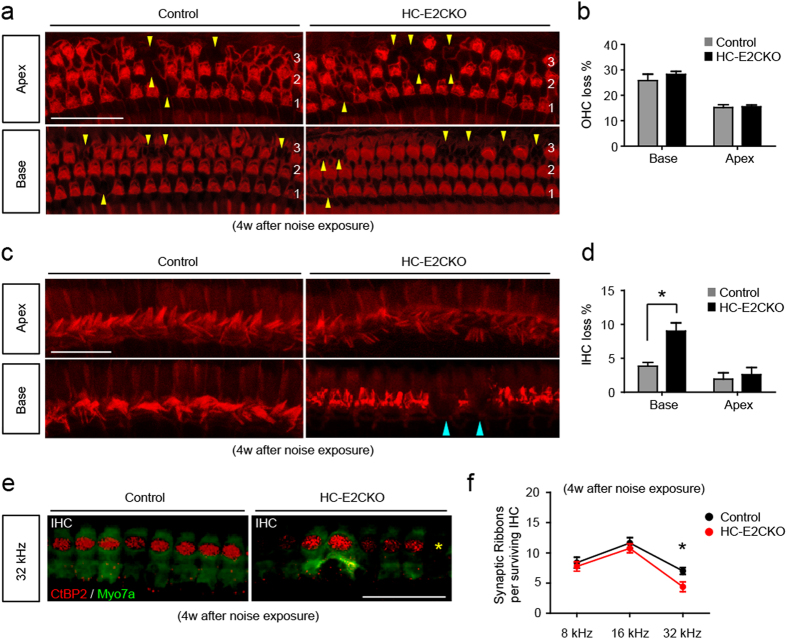
HC-E2CKO mice showed more IHC loss compared with controls after noise exposure. (**a**) Images of OHCs in control and HC-E2CKO mice at 4 weeks after noise exposure. HC-E2CKO mice had OHCs loss (yellow) comparable to control mice. 1, 2, 3: 1^st^, 2nd, and 3rd rows of OHCs. Scale bar, 50 μm. (**b**) The ratio of missing to whole OHCs is expressed as a percentage. No significant differences were observed in OHCs loss between the groups (n = 6 in each group). (**c**) Images of IHCs in control and HC-E2CKO mice at 4 weeks after noise exposure. In the base, HC-E2CKO mice had more IHC loss (blue) compared to control mice. Scale bar, 25 μm. (**d**) The ratio of missing to whole IHCs is expressed as a percentage. A significant loss of IHCs was observed at the base in HC-E2CKO mice compared with control mice (n = 6 in each group). (**e**) Whole mount preparations were stained with myosin7a (green) and CtBP2 (red). HC-E2CKO mice showed loss (yellow asterisks) of IHCs and synaptic ribbons. IHC, inner hair cell; Myo7a, myosin7a. Scale bar, 20 μm. (**f**) The number of synaptic ribbons per surviving IHC at 32 kHz was significantly decreased in HC-E2CKO mice (n = 4 in each group). Error bars show SE. (Mann-Whitney U-test, *p < 0.05).

To compare HC loss 4 weeks after noise exposure, surface preparations of cochleae from HC-E2CKO and control mice (n = 6 for each group) were stained for phalloidin. The number of OHCs that had been lost was not changed in HC-E2CKO mice compared with the control group at both the base and apex (Fig. 6a,b). On the other hand, the number of IHCs that had been lost in the base was increased in HC-E2CKO mice compared with the control group (Fig. 6c). Since the base corresponds to the position that codes for high frequency in the cochlear duct, these findings are consistent with the result that the ABR threshold was increased in the high frequency range in HC-E2CKO mice. Our quantification indicated that, in the base, HC-E2CKO had significantly fewer IHCs than the control group (Fig. 6d; Mann-Whitney U-test, p = 0.012). On the other hand, in the apex, no significant difference in the extent of IHCs loss was observed between HC-E2CKO and control mice. We observed conspicuous actin cables in regions where HCs were missing in both control and HC-E2CKO mice, indicating that a scar had been formed at these sites (Fig. 6a,c).

Furthermore, we investigated whether the number of synaptic ribbons was changed in HC-E2CKO mice after noise exposure compared with control mice (Fig. 6e). In the 32 kHz region, noise exposure significantly decreased the number of synaptic ribbons per IHC in HC-E2CKO mice compared with the control group (Fig. 6f; Mann-Whitney U-test, p = 0.019; n = 4 for each group), although no significant difference was observed in 8 and 16 kHz regions. Collectively, our data indicate that, in response to acoustic trauma, ERK2 plays an important role in mediating the survival of IHCs and synaptic ribbons.

## Discussion

In the current study, using a conditional genetic approach, we revealed that abrogation of ERK2 specifically in mouse HCs increased susceptibility to acoustic trauma. This finding suggests that activation of ERKs in HCs after noise exposure might have important protective effects against NIHL. Our results are consistent with previous reports that have implicated that active forms of ERK act as prosurvival factors^12^,^14^ and that the basal level of ERK activation in HCs is important for their survival^13^. Contrary to our results, it has been reported that MEK1/2 inhibition significantly reduces IHC death caused by 8 h of neomycin treatment, suggesting that the activation of ERK might act to promote HC death^11^. Although the reason for the difference is unclear, one possible explanation is that the ERK signaling pathway plays a complex role in the regulation of distinct cellular responses. Indeed, the cellular functions that are regulated by ERK seem to depend on the cell type, stimulus, duration, and strength of kinase activity. For instance, phosphorylation levels of ERK1/2 are different after temporary or permanent hearing loss^7^. Thus, additional studies are required to understand how ERK activation is regulated in the cochlea after acoustic trauma.

Our ABR results suggested that HC-E2CKO mice had hearing comparable to control mice (Fig. 5b). Although mice were at 8-weeks of age, the baseline thresholds at 32 kHz in HC-E2CKO (50 dB SPL) and control mice (57 dB SPL) were slightly higher compared with previous study^23^. One explanation for this may be attributed to the fact that we used mice with a C57BL/6J background, which are known to show threshold elevation at higher frequencies^24^,^25^,^26^.

Furthermore, acoustic overstimulation resulted in a relatively rare situation whereby HC-E2CKO mice exhibited a prominent loss of IHCs in addition to OHC loss, while control mice exhibited only loss of OHCs. The severe loss of IHCs and synaptic ribbons in HC-E2CKO mice after noise exposure suggests that deletion of the *Erk2* gene may underlie some forms of “auditory neuropathy,” a clinical diagnosis characterized by IHC dysfunction and synaptic ribbon loss^27^,^28^. In most cases of SNHL, whether due to noise^29^ or ototoxic drugs^30^, OHCs tend to be the most vulnerable elements in the inner ear. We found that the immunoreactive signal of p-ERK1/2 was increased in the OHCs and DCs in HC-E2CKO mice after noise exposure. As for IHCs, we could not detect ERK1 signals in both control and HC-E2CKO mice (Fig. 3a). On the other hand, we found that the p-ERK1/2 signal was detected in IHCs from control mice but not in HC-E2CKO mice after noise exposure (Figs 1c and 4). Taken together, these results suggest that ERK2 is mainly phosphorylated in IHCs after noise exposure. Because ERK2 was deleted in the OHCs as well as IHCs of HC-E2CKO mice, the increased p-ERK signal in the OHCs of HC-E2CKO mice after noise exposure might indicate the presence of p-ERK1 in the OHCs. Thus, our results indicate that ERK1 in the OHCs was phosphorylated after noise exposure, although this does not exclude the possibility of ERK2 phosphorylation in the OHCs of control mice after noise exposure. Therefore, our finding suggests that ERK1 may compensate for the lack of ERK2 in the OHCs of HC-E2CKO mice. In contrast, the same compensatory mechanism might not be available in IHCs due to the lack of immunohistochemistry-detectable ERK1. Consistent with our result, it was recently reported that IGF-1 protects cochlear HCs from the damage caused by noise and neomycin and that only ERK1 may be involved in the protection of OHCs against neomycin^31^. Furthermore, they suggested that ERK2, but not ERK1, may be important for the survival of IHCs^31^ consistent with our results.

In the current study, we used a *pou4f3*–Cre mouse line to delete the targeted gene. ERK1/2 has been reported to be necessary for cochlear development^32^. Although *pou4f3*–cre mice express Cre recombinase beginning at embryonic day 12.5 (E12.5) in vestibular HCs and in cochlear basal HCs at E14.5^33^, HC-E2CKO mice in the current study showed normal cochlear morphology and a full complement of both OHCs and IHCs. This finding suggests that ERK2 might be dispensable for cochlear development. Alternatively, ERK2 disruption might be compensated for by ERK1 in HC-E2CKO mice. Moreover, in this study, ERK2 immunoreactivity in HC-E2CKO mice was reduced not only in HCs but also SCs, including DCs. Cochlear SCs participate in HC elimination and scar formation after noise exposure by sealing the reticular lamina, a barrier between the endolymph and perilymph^34^. This scarring process is important to preserve remaining HCs and hearing^34^. Our results indicated that there was not a significant impairment in scar formation of the HC region of HC-E2CKO mice after noise exposure, suggesting that ERK2 may not be important for scar formation after noise exposure.

Erk2 is located on human chromosome 22q11.2, the deletion of which leads to 22q11.2 deletion syndrome (22q11.2DS)^35^,^36^. Typical findings in patients with 22q11DS include auditory impairment and conductive hearing loss (~45% of patients)^35^. Hearing loss in these patients is thought to be caused by malformations of the middle ear or otitis media^35^,^36^. Indeed, chronic or recurrent otitis media has been reported in 52% of patients with 22q11DS^37^. On the other hand, about 10% of patients with 22q11DS experience SNHL^35^, although its underlying mechanism remains unknown. In our study, HC-E2CKO mice did not exhibit any obvious abnormalities of the middle ear although they exhibited severe SNHL after noise exposure. Thus, hypofunction of ERK2 might contribute to SNHL in 22q11DS upon acoustic injury.

Additional experiments are necessary to address the other mechanisms underlying noise-induced HC death and functional relationship between ERK1 and ERK2, including the analysis of ERK1-knockout mice. Further study will also aid our understanding of the individual roles of ERK1 and ERK2 in the cochlea.

## Materials and Methods

### Mice

All experiments were conducted according to the institutional ethical guidelines for animal experiments and the safety guidelines for gene manipulation experiments of the National Defense Medical College (Tokorozawa, Saitama, Japan). These experiments were approved by the Committee for Animal Research at the National Defense Medical College. Animals were maintained on a 12 h light-dark cycle (lights on from 7:00 A.M. to 7:00 P.M.) with room temperature at 21 ± 1 °C. Mice had ad libitum access to water and food.

To obtain HC-E2CKO mice, *Erk2* floxed mice^38^ were crossed with *pou4f3-cre* transgenic mice^39^,^40^, which were maintained on the same background (C57BL/6J). All mice used in this study were age-matched male littermates (8 weeks of age).

### Histopathological analysis

Mice were anesthetized by an intraperitoneal injection of ketamine (50 mg/kg) and medetomidine (1.0 mg/kg) and perfused with 4% paraformaldehyde (PFA). Cochleae were then removed and kept in the fixative overnight. After decalcification with 0.1 M ethylenediaminetetraacetic acid (EDTA) in phosphate-buffered saline (PBS) for 10 days at 4 °C, sections were dehydrated, embedded in paraffin, and sliced into 10-μm sections. Sections were then deparaffinized, stained with hematoxylin-eosin, and viewed under light microscopy (BX51; OLYMPUS, Japan).

### Immunohistochemistry

Paraffin-embedded sections were deparaffinized and immersed in unmasking solution (Vector H3300; Vector Laboratories) for antigenic retrieval and heated in an autoclave (121 °C) for 5 min. Sections were then incubated with a non-specific blocking reagent (Dako) for 1 h to block any non-specific reactions, followed by overnight incubation at 4 °C with one of the following primary antibodies: anti-ERK1 (Invitrogen, 13–8600), anti-ERK2 (Cell Signaling, #9108), anti-p-ERK1/2 (Cell Signaling, #9101), anti-myosin VIIa (Proteus Biosciences, 25–6790), or anti-CtBP2 (BD Transductions, 612044) diluted in an antibody diluent (DAKO). Sections were washed thrice in PBS and incubated with a corresponding secondary antibody (Alexa Fluor 488 or 546, IgG, Invitrogen) diluted in an antibody diluent (DAKO). After rinsing in PBS, NSE was mounted onto slides with an antifade mounting medium (VECTASHIELD with DAPI; Vector Laboratories, Burlingame, CA, USA). DAPI labeling was then used to identify condensed HC nuclei. Images of immunolabeled specimens were obtained by confocal fluorescence microscopy using a Nikon C2 system (Nikon, Tokyo, Japan).

### Noise exposure

Mice (8 weeks of age) anesthetized with ketamine/medetomidine were exposed to two octave-bands of noise (2–8 kHz) at 121 dB SPL for 5 h in a ventilated sound exposure chamber. The sound chamber was fitted with speakers (Model 2380A; JBL, Northridge, CA, USA) driven by a noise generator (DANAC-31; Dana Japan, Tokyo, Japan) and power amplifier (D-45; Crown International, Elkhart, IN, USA). Sound levels were calibrated (Type 6224 precision sound level meter; Aco Instruments, Tokyo, Japan) at multiple locations within the sound chamber to ensure uniformity of the stimulus.

### Preparation of protein extracts

Immediately after decapitation, cochleae were dissected by removing lateral wall bones in PBS at 4 °C, and the surrounding LW tissues, including the stria vascularis, were carefully collected. The remaining cochlear tissues were carefully divided into two portions: NSE and MO. The obtained cochlear tissues were homogenized in buffer containing 50 μL of 20 mM Tris-HCl (pH 7.4), 2 mM EDTA, Complete Inhibitor Mix (Roche, Indianapolis, IN), and PhosSTOP phosphatase inhibitors (Roche, Indianapolis, IN). After homogenization, the homogenate was centrifuged at 15000 *g* for 30 min at 4 °C. The supernatant solutions were then separated and stored at −80 °C until use. The amount of protein concentration in each sample was measured using a bicinchoninic acid protein assay kit (Pierce, Rockford, IL).

### Western blotting

Western blot analysis of forebrain was performed as previously described^41^. Briefly, the proteins in the SDS-PAGE gel were transferred onto an Immobilon-P membrane (Millipore, Bedford, MA). Blots were then immunoreacted with anti-ERK1 (Invitrogen, 13–8600), anti-ERK2 (Cell Signaling, #9108), anti-p-ERK1/2 (Cell Signaling, #9101) and anti-β-actin (Cell Signaling, #4967) antibodies diluted in 5% skim milk, and protein bands were visualized using a chemiluminescence detection system (Super Signal West Dura [Pierce, Rockford, IL, USA] or ECL plus [GE Healthcare, Little Chalfont, UK]). Signals in the immunoblots were analyzed by a LAS4000 digital imaging system (Fujifilm, Tokyo, Japan).

### Auditory brainstem response

The ABR was measured using a single recorder (Syntax 1200; NEC, Tokyo, Japan) before and immediately after noise exposure, and then 4 weeks after noise exposure. Following anesthesia, stainless steel needle electrodes were placed subcutaneously at the vertex and ventrolateral to the left and right ears for recording. Tone burst stimuli with a 1-ms rise/fall time at frequencies of 4, 8, 12, 16, 20, and 32 kHz were generated. The burst amplitudes were specified by a sound generator and attenuated by a real-time processor and programmable attenuator (RP2.1 and PA5; Tucker-Davis Technologies, Alachua, FL, USA). Sound stimuli were produced by a coupler-type speaker (ES1spc; Bio Research Center, Nagoya, Japan). ABR waveforms were recorded in 10−dB SPL intervals down from the maximum amplitude until no waveform could be visualized.

### Quantitative assessment of hair cell loss

Animals were decapitated 4 weeks after noise exposure. The bone near the apex was removed and the round and oval windows of the inner ear were opened, followed by gentle local perfusion with 2 × 1 mL 4% PFA in 0.1 M PBS (pH 7.4). The tissues were then preserved overnight in the fixative. Cochleae were dissected by removing the lateral wall bones, lateral wall tissues, and tectorial membranes. After several washings with PBS, the remaining parts of the cochleae were incubated in 0.3% Triton X-100 in PBS for 5 min and washed 3 times with PBS. The NSE was stained for F-actin with 1% rhodamine phalloidin (Invitrogen, Carlsbad, CA, USA) for 60 min to outline HCs and their stereocilia for quantitative assessment. After several washings with PBS, cochleae were dissected and mounted for surface preparation. The tissues were observed under confocal fluorescence microscopy using the Nikon C2 system (Nikon, Tokyo, Japan), and the numbers of missing OHCs and IHCs in the base and apex of the cochleae were counted. The ratio of missing-to-whole HCs was expressed as a percentage.

### Quantitative assessment of synaptic ribbons

Assessment of synaptic ribbons was performed using a custom-written ImageJ plugin (available at http://www.masseyeandear.org/research/otolaryngology/investigators/laboratories/eaton-peabody-laboratories/epl-histology-resources/imagej-plugin-for-cochlear-frequency-mapping-in-whole-mounts). Cochlear lengths were obtained for each case, and a cochlear frequency map was computed to precisely localize IHCs from the 8, 16, and 32 kHz regions. Confocal z-stacks of these three regions from each ear were obtained using a Nikon C2 system (Nikon, Tokyo, Japan) to span the entire synaptic pole of IHCs in the z-dimension, with a z-step-size of 0.25 μm. The number of synaptic ribbons per IHC in a 100-μm range was counted in each of the three frequency-specific regions.

### Data analysis

All quantitative analyses were performed by an investigator who was blind to genotypes during experimentation and data analysis. All data values are presented as means ± standard error (SE). Relative levels of ERK phosphorylation, ABR threshold shifts, percentage of missing HCs, and the number of synaptic ribbons per IHC were evaluated using Mann-Whitney U-tests. A p-value of less than 0.05 was considered statistically significant.

## Additional Information

**How to cite this article**: Kurioka, T. *et al.* ERK2 mediates inner hair cell survival and decreases susceptibility to noise-induced hearing loss. *Sci. Rep.* **5**, 16839; doi: 10.1038/srep16839 (2015).
